# New geographic location data on the occurrence and abundance of carrion insects of forensic interest

**DOI:** 10.1007/s12024-024-00780-0

**Published:** 2024-01-30

**Authors:** Beryl Morris

**Affiliations:** University of Queensland, TERN, St Lucia, 4072 Australia

**Keywords:** Forensic entomology, Carrion insects, Standard protocols, Trapping

## Abstract

The world’s forensic entomologists have much in common. They face similar research challenges, apply the same scientific methodology, study the same kinds of evidence, and access global research databases. Nevertheless, some regional heterogeneity inevitably exists. For most countries, and particularly those that have complex and diverse ecosystems, the current priority is to stimulate use of forensic entomology by establishing open access databases with time series data using standardised protocols for occurrence, abundance, distribution, niche preferences, life cycle, and identification characteristics for the key regional species that may be encountered in forensic entomology cases. Even in countries where forensic entomology is routinely used as a tool in reconstructing the history of corpses found on crime scenes in accordance with principles found in the rapidly developing body of literature, there is still much to learn about forensically useful insects. Examples of regional gaps include the taxonomy of lesser-known carrion insect species, seasonal occurrence of carrion species in the country’s various geographical regions, and rates of development of the local species modeled in the many site situations of forensic interest. The first published study of carrion insects found in Athens, Greece published in this journal is an example of entomologists in a region taking the necessary first step towards establishing baseline data about native and introduced species and hence, physiological and behavioural responses to local environmental conditions, including life cycles and likelihood of occurrence or absence in the region.

Forensic entomology has been a useful tool for crime scene investigators for more than a century in the Western world. Even so, there is still much to learn about forensically useful insects. One critical gap in our knowledge is the seasonal occurrence in various geographical regions of those insect taxa predictably associated with the decomposition stages of carcases, carrion and corpses. Fortunately, research worldwide continues to fill the information gaps with the aim of further improving the credibility and use of entomological evidence in forensic science.

The latest welcome contribution to local baseline information on species occurrence of forensically useful insects comes from “A preliminary study of insects in Greece and their attraction to three animal baits: A forensic entomology perspective,” the first such occurrence study of insects attracted to decomposing animal material to be published from Greece. The study had 2 objectives, the first of which was to record the composition of dipteran (flies) and coleopteran (beetles) fauna found in baited traps at the Agricultural University of Athens. With mixed agricultural practices at the farm, covering both plant and animal activities, there was a high probability of insects related to decomposition already being in the area and hence an ideal choice for the location of the experiments involving 3 bait types in each of 2 types of traps deployed across 3 localities on the farm. The second objective of the study was to examine the preferred bait choice of the insects collected.

Studying insect occurrence by using baited traps, as in the experiment above, seeks to make use of the way insects perceive the world, which is to rely almost exclusively on chemicals they detect from their environment, using olfactory organs located primarily on the antennae [1]. The insects use the detected chemical signals to navigate through their environment and quickly process the information in an odour plume coming from a source, which in the mentioned study, was an array of baited traps. From the sequence in which insects typically arrive at a bait or carcase, it is evident that even the state of decomposition of a carcase or bait is revealed through the volatiles released. For application to forensic entomology, it is important that baited traps act as a reasonable proxy for a decomposing cadaver or carcase.

Based on a scan of the literature, there have been a growing number of studies which have used baited traps to study composition of carrion insect fauna in new locations. Among their advantages, the baited traps are relatively cheap, do not require complicated ethics approvals and can be deployed by one person in locations continuously accessible to the researcher; in general, they are not disturbed by scavengers in the same manner that carcases are, and placement and sampling of the traps plus analysis can be conveniently completed within the range of timetables applicable to formal research study programs in higher educational institutions.

However, there have been sufficient studies of carrion insects over the last century to show that carrion insect fauna and rates of decomposition of food sources vary greatly according to season, latitude, altitude, nature of food source and many other factors. The different outcomes of experiments around the world show that small differences in experimental design can significantly alter the outcome, while every environmental detail and physical feature of the carcasses or food source may contribute to the resulting fauna and its succession and development. For example, flies from the Calliphoridae family respond differently to butchered meat compared to natural carrion [2]. Indeed, knowledge of carrion insects in a geographic area does not necessarily provide confidence that the results of experimental studies can be duplicated [3, 4]. At present, few studies of carrion insects span more than one season and are thus clearly limited in their applications to forensic cases.

Some authors have expressed concern about whether trapping data truly reflect the abundance and pattern of carrion insects in an area at any particular time of the year. This is because it is not directly possible to correct for the effect of weather on seasonal abundance, and hence, the possibility exists that the observed presence, absence and abundance of flies reflect the weather occurring during that specific survey [5]. Temperature, cloudiness, humidity, wind, time of day and total carrion insect population of an area are also potential influencers of the abundance and activity of carrion insects, and in at least the case of species of Calliphoridae, habitat preferences may cause differences in abundances within short distances [2].

Despite awareness of the methodological limitations of traps, regretfully, there is no agreed standard protocol yet for undertaking survey studies using baited traps. For uptake of outcomes into mainstream forensic entomology from the experiments on carrion insect fauna composition at specific locations, experimenters would optimally use agreed protocols in their local studies which rely on clear and proven methods (built upon previous method(s) where appropriate) to consistently record carrion fauna occurrence and abundance, and over time, the monitoring exercises would be repeated at the same locations in different seasons and years to measure change in relation to many environmental and social variables of interest. Widely adopted standard protocols over many locations would address the limitations in generalising about carrion insect distribution over space and seasonal abundance over time that makes current quantification and prediction of impacts of environmental and social change on carrion insects difficult. At present, without standard protocols, we are left with occurrence date measured or estimated using disparate and often incompatible methods, particularly over a range of geographic scales, and inefficiencies in data provision and analysis.

## Conclusion

It is important from a forensic science perspective that gaps in knowledge be filled about the regional occurrence and abundance of carrion insect fauna, as is the case with the first published study of carrion insects found in Athens, Greece. Such studies are a valuable step towards establishing baseline data about native and introduced species and hence, physiological and behavioural responses to local environmental conditions, including life cycles and likelihood of occurrence or absence in a region. With repeat surveys, baseline data are also important for measuring and predicting changes over time and space of the diversity and abundance of carrion insect fauna and, eventually, support development of a forensic entomology database from which to support judicial systems such as that in Greece, with expert opinions concerning, for instance, estimates of minimum time since death in cases involving homicide.

Although all data on carrion fauna biodiversity has value, each new study on the carrion insect ecology of a geographical region highlights the need for agreed standard protocols from the forensic entomology community for carrying out experiments using baited traps. Such standardization of methods is essential if we are to move to time series data that not only spans more than one season, but which also allows modelling and prediction of patterns of occurrence and abundance across seasons and geographic regions. With such an approach, not only will information gaps on the insect composition be filled but the credibility of forensic evidence within and across jurisdictions will be commensurately improved.
